# Assessment of the Effect on Thromboprophylaxis with Multifaceted Quality Improvement Intervention based on Clinical Decision Support System in Hospitalized Patients: A Pilot Study

**DOI:** 10.3390/jcm11174997

**Published:** 2022-08-25

**Authors:** Qian Gao, Kaiyuan Zhen, Lei Xia, Wei Wang, Yaping Xu, Chaozeng Si, Zhu Zhang, Fen Dong, Jieping Lei, Peiran Yang, Jixiang Liu, Ziyi Sun, Tieshan Zhang, Jun Wan, Wanmu Xie, Peng Liu, Cunbo Jia, Zhenguo Zhai, Chen Wang

**Affiliations:** 1 Department of Pulmonary and Critical Care Medicine, Center of Respiratory Medicine, China-Japan Friendship Hospital, Beijing 100029, China; 2 Institute of Respiratory Medicine, Chinese Academy of Medical Sciences, Beijing 100029, China; 3 National Clinical Research Center for Respiratory Diseases, Beijing 100029, China; 4 Peking University China-Japan Friendship School of Clinical Medicine, Beijing 100029, China; 5 Medical Affairs Department of China-Japan Friendship Hospital, Beijing 100029, China; 6 Department of Nursing, China-Japan Friendship Hospital, Beijing 100029, China; 7 Department of Information Management, China-Japan Friendship Hospital, Beijing 100029, China; 8 Institute of Clinical Medical Sciences, China-Japan Friendship Hospital, Beijing 100029, China; 9 Department of Physiology, Institute of Basic Medical Sciences, Chinese Academy of Medical Sciences, Peking Union Medical College, Beijing 100730, China; 10 Chinese Academy of Medical Sciences and Peking Union Medical College, Beijing 100730, China; 11 China-Japan Friendship Hospital, Beijing 100029, China; 12 Department of Oncology, Beijing Electric Power Hospital, Capital Medical University, Beijing 100073, China; 13 Department of Pulmonary and Critical Care Medicine, Beijing Anzhen Hospital, Capital Medical University, Beijing 100029, China; 14 Beijing Institute of Heart, Lung and Blood Vessel Diseases, Beijing 100029, China; 15 Department of Cardiovascular Surgery, China-Japan Friendship Hospital, Beijing 100029, China

**Keywords:** Venous thromboembolism, quality improvement, VTE prophylaxis

## Abstract

Background: To explore the feasibility and effectiveness of multifaceted quality improvement intervention based on the clinical decision support system (CDSS) in VTE prophylaxis in hospitalized patients. Methods: A randomized, department-based clinical trial was conducted in the department of respiratory and critical care medicine, orthopedic, and general surgery wards. Patients aged ≥18 years, without VTE in admission, were allocated to the intervention group and received regular care combined with multifaceted quality improvement intervention based on CDSS during hospitalization. VTE prophylaxis rate and the occurrence of hospital-associated VTE events were analyzed as primary and secondary outcomes. Results: A total of 3644 eligible residents were enrolled in this trial. With the implementation of the multifaceted quality improvement intervention based on the CDSS, the VTE prophylaxis rate of the intervention group increased from 22.93% to 34.56% (*p* < 0.001), and the incidence of HA-VTE events increased from 0.49% to 1.00% (*p* = 0.366). In the nonintervention group, the VTE prophylaxis rate increased from 24.49% to 27.90% (*p* = 0.091), and the incidence of HA-VTE events increased from 0.47% to 2.02% (*p* = 0.001). Conclusions: Multifaceted quality improvement intervention based on the CDSS strategy is feasible and expected to facilitate implementation of the recommended VTE prophylaxis strategies and reduce the incidence of HA-VTE in hospital. However, it is necessary to conduct more multicenter clinical trials in the future to provide more reliable real-world evidence.

## 1. Introduction

Venous thromboembolism (VTE) includes pulmonary thromboembolism (PE) and deep vein thrombosis (DVT). The estimated incidence of VTE is 115–269 per 100,000 globally, and the mortality rate is 6.8–32.3 per 100,000 [1,2]. The majority (55–60%) of VTE events occur during hospitalization or 90 days after discharge, which are considered as hospital-associated VTE (HA-VTE) [3]. As a major preventable inpatient adverse event, the incidence of VTE can be effectively reduced by standardized preventive measures such as the prophylactic use of anticoagulants and mechanical prophylaxis [4,5,6].

VTE prophylaxis is the key measure in reducing VTE incidence and VTE-related mortality and morbidity in medical and surgical inpatients. The guidelines in China recommend that clinicians should adopt various individualized prophylaxis strategies based on adequate assessment of VTE risk and bleeding risk and adjust prophylaxis strategies based on dynamic assessment results [7,8]. The American College of Chest Physicians (ACCP) guidelines for thromboprophylaxis have clearly stated the importance of anticoagulant prophylaxis and mechanical prophylaxis [9]. Several academic institutions also have developed guidelines and recommendations on VTE prophylaxis [6,10,11,12].

Many initiatives have been taken in several countries to prevent VTE in hospitals with impressive results: In 2010, the National Health Service (NHS) launched the National Venous Thromboembolism Prophylaxis Programme. The National Institute for Health and Care Research published guidelines for inpatient VTE prophylaxis. Through the use of mandatory VTE risk assessment tools, the VTE risk assessment rate increased rapidly from 50% in 2010 to 90% at the beginning of Q4 2011 and has remained above 95% since 2013, achieving a 10.8% reduction in VTE-related mortality over the same period [11]. In 2008, the Agency for Healthcare Research and Quality Management (AHRQ) published guidelines for reducing HA-VTE, which were updated again in 2016 [6,13]. In 2012, the New Zealand Health Quality & Safety Commission also released a national policy document to prevent HA-VTE [14].

However, there remains a gap between the recommended preventive and measures clinical practice. Between 2007 and 2016, the incidence of VTE in Chinese inpatients increased from 3.2 to 17.5 per 100,000, while the in-hospital VTE-related mortality rate decreased from 4.7% to 2.1% [15]. At the same time, the DissolVE-2 study showed that VTE prophylaxis rates in China were severely underrepresented at only 19.0% and 9.3% among surgical and medical inpatients, respectively, with even lower rates of appropriate prophylaxis [16]. This result was much lower than the 40–60% VTE prophylaxis rates reported by a global multicenter study in 2008 [17]. The gap between the increasing incidence and the highly inadequate prophylaxis highlights the need to strengthen VTE prophylaxis, which has become an urgent clinical issue. Recent advances in machines learning and deep learning based on the increased availability of clinical data have stimulated new interest in a computerized clinical decision support system (CDSS) [18]. The CDSS shows great potential in improving health care, improving patient safety, and reducing medical costs. To facilitate implementation of appropriate thromboprophylaxis, the Chinese Prevention Strategy for Venous Thromboembolism (CHIPS-VTE) study network developed a system-wide multifaceted quality improvement strategy based on the CDSS [19]. This single-center study aims to explore the feasibility and effectiveness of multifaceted quality improvement intervention based on CDSS in VTE prophylaxis in hospitalized patients.

## 2. Methods

### 2.1. Study Design and Participants

In this pilot study, a single-center, department-based, cluster randomized trial was conducted at the China–Japan Friendship Hospital by comparing VTE prevention-related performance between departments applying multifaceted quality improvement intervention and those applying regular care. A total of ten medical or surgical units from the departments of Pulmonary and Critical Care Medicine, Orthopedics, and General Surgery participated in this study. 

The study included two periods: the baseline period was from 1 October 2019 to 31 December 2019 and the intervention period from 1 April 2020 to 30 June 2020. Patient information was not collected from 1 January to 31 March 2020 due to the COVID-19 pandemic, during which the clinical care of inpatients was not representative of standard clinical practice.

Adult patients with a length-of-stay of more than 3 days or receiving surgery under anesthesia were considered to be included. Patients with hospitalization of less than 3 days without receiving surgery, diagnosed with VTE before admission or with community-acquired VTE after admission, with acute myocardial infarction (AMI), atrial fibrillation (AF), acute stroke (AS), mechanical heart valve replacement, extracorporeal membrane oxygenation (ECMO), or dialysis in admission were considered to be excluded. 

### 2.2. Cluster Randomization and Intervention

We randomly divided medical or surgical units, based on the prophylaxis rates of each participating unit at the baseline period, into intervention groups and nonintervention groups, to reduce the contamination bias within the same clinical unit. Both groups were asked to include three different units of Orthopedic, Respiratory and Critical Care Medicine, and General Surgery. 

**Figure 1:**
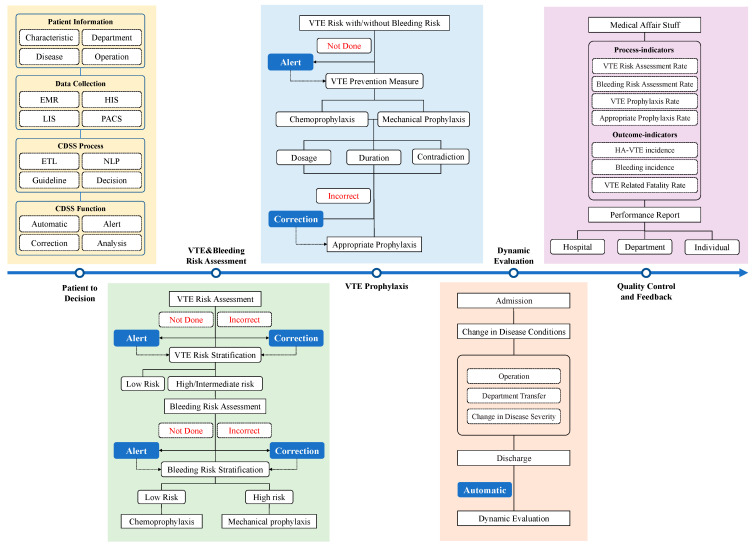
Process of multifaceted quality improvement intervention based on CDSS. EMR: electronic medical record; HIS: hospital information system; LIS: laboratory information system; PACS: picture archiving and communication system; ETL: extract–transform–load; NLP: natural language processing.

The intervention group was subjected to multifaceted quality improvement intervention based on the CDSS, which included the application of the CDSS with electronic alertness assistance for VTE prophylaxis, dynamic VTE risk assessment, and prophylaxis. The CDSS has four fundamental functions: automatic, reminder, correction, and quality control analysis. The CDSS can automatically collect and analyze patients’ information from various information platforms in-hospital such as electronic medical record (EMR), hospital information system (HIS), laboratory information system (LIS), picture archiving and communication system (PACS), etc. Through extract–transform–load (ETL), natural language processing (NLP), and other technologies, timely and accurate reminders and supporting decisions were automatically provided to clinicians according to clinical diagnosis and treatment guidelines (Figure 1). The CDSS could automatically analyze the patient medical records to assist medical staff in making decisions on VTE risk assessment and appropriate prophylaxis, with error correction and reminder features. Electronic alertness could automatically and actively remind medical staff to complete the VTE risk assessment and prophylaxis in a pop-up window in the electronic medical record system when they failed to complete the risk assessment or prophylaxis. If clinicians disagreed with the advice made by the CDSS, they could refuse the decisions with plausible explanations.

We established a multidisciplinary VTE prevention expert committee to formulate the VTE prevention process of the hospital. The units assigned to the nonintervention group implemented the hospital’s current VTE prophylaxis measures which is according to the guidelines suggesting that doctors confirm the results of risk assessment conducted by nurses and make a prophylaxis order without additional interventions such as mandatory reminders and corrections.

### 2.3. Statistical Analysis

The primary outcome was the implementation of any VTE prophylaxis measurements in hospitalized patients. For patients at intermediate or high risk of VTE, VTE prophylaxis must be used if there were no other relevant contraindications; if there was a high risk of bleeding, mechanical prophylaxis should be applied; if there was no high risk of bleeding, pharmacological prophylaxis or pharmacological prophylaxis combined with mechanical prophylaxis should be applied.

The secondary outcome was HA-VTE events in hospitalization, which was determined by as follows: (1) admission diagnosis without VTE and discharge diagnosis of new-onset VTE, with manual verification; (2) patients who already had VTE or were already receiving anticoagulation for other diseases at admission were not included.

Normally distributed measurement data are presented as mean ± standard deviation, and an independent sample *t*-test was used for comparison between the two groups. Non-normally distributed measurement data are presented as median (upper and lower quartiles), and count data are expressed as absolute numbers (N) and percentages (%). For comparison of differences between groups, Wilcoxon rank sum test was used for non-normally distributed data, and chi-square test for qualitative data. A two-tailed *p* < 0.05 was regarded as statistically significant. SPSS 24.0 was used for statistical analysis in this study.

## 3. Results

### 3.1. Patient Characteristics

**Figure 2:**
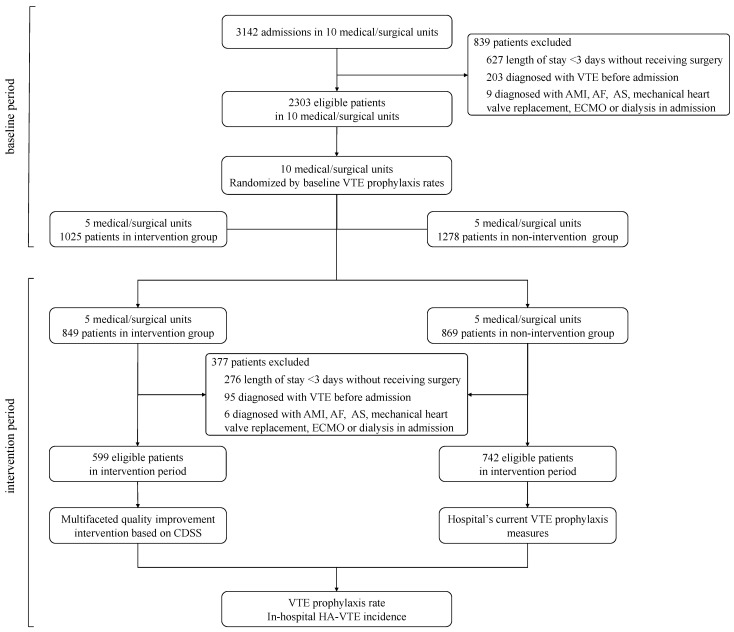
Recruitment process and flow through study. LOS: length of stay; AMI: acute myocardial infarction; AF: atrial fibrillation; AS: acute stroke; ECMO: extracorporeal membrane oxygenation.

A total of 3644 eligible patients were enrolled in the study. Out of that total, 1624 cases were included in the intervention group, of which 1025 were in the baseline period, and 599 were in the intervention period; 2020 cases were included in the nonintervention group, of which 1278 were in the baseline period, and 742 were in the intervention period. The study flow is shown in Figure 2. There was no statistical difference between the two groups in terms of age more than 40 years and mean length-of-stay (*p* > 0.05). However, there were more male patients in the intervention group and a higher proportion of inpatients aged under 40 and between 61 and 74 in the nonintervention group. A comparison between patients in the two groups during each period is shown in Table 1.

### 3.2. Improvement in VTE Risk Assessment

A total of 3374 (92.59%) patients were given VTE risk assessment, including 2167 (94.09%) patients in the baseline period and 1207 (90.00%) in the intervention period. 

**Figure 3:**
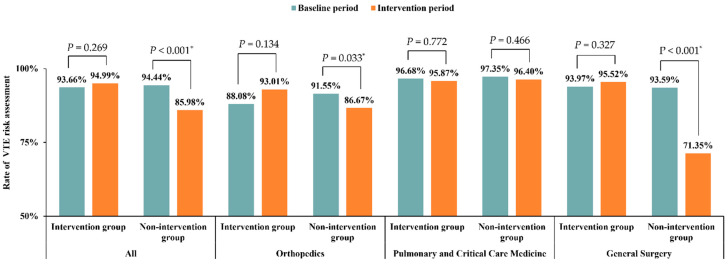
Improvement in VTE risk assessment in different departments. * *p* < 0.05.

For patients of the intervention group, the VTE risk assessment rates were slightly increased from 93.66% in the baseline period to 94.99% in the intervention period (*p* = 0.269). However, the VTE risk assessment rates were found decreased in the nonintervention group from 93.89% in the baseline period to 83.83% in the intervention period (*p* < 0.001), as shown in Figure 3. Among patients who received the VTE risk assessment, 1927 (57.11%) patients were stratified into intermediate or high risk of VTE.

The rate of VTE risk assessment remained stable in the intervention group in the departments of Orthopedics, Pulmonary and Critical Care Medicine, and General Surgery across the study (Figure 3). However, significant decreases were found in nonintervention group in the departments of Orthopedics and General Surgery (*p* < 0.001 for both). 

### 3.3. Improvement in VTE Prophylaxis

**Figure 4:**
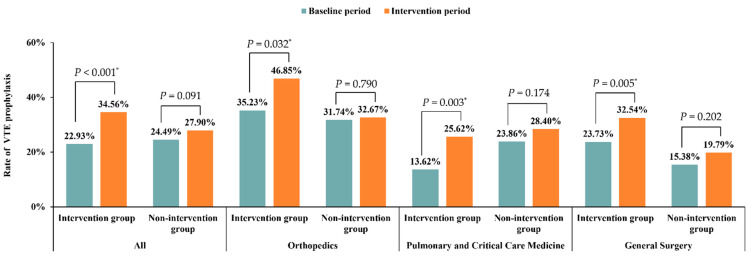
Improvement of VTE prophylaxis in different departments. * *p* < 0.05.

As for VTE prophylaxis, 962 (26.40%) patients were given VTE prophylaxis in the study. No statistical differences between intervention and nonintervention groups were found in the baseline period (22.93% vs. 24.49%, *p* = 0.091) (Table 1). Although patients in both groups showed a poor rate of VTE prophylaxis, a significant increase was found in the intervention group from the baseline period to the intervention period (22.93% to 34.56%, *p* < 0.001). In contrast, in the nonintervention group, the VTE prophylaxis rate changed from 21.65% in the baseline period to 27.16% in the intervention period (*p* = 0.269), as shown in Figure 4. There was also a statistically significant difference between the two groups in the intervention period (27.90% vs. 34.56%, *p* = 0.009). 

In the intervention group, significant improvements of VTE prophylaxis were observed in the departments of Orthopedics, Pulmonary and Critical Care Medicine, and General Surgery. The corresponding *p* values were 0.032, 0.003, and 0.005, respectively. No statistical differences were found in nonintervention group in any department. The corresponding *p* values were 0.790, 0.174, and 0.202, respectively (Figure 4).

Among patients receiving VTE prophylaxis, 952 (98.96%) patients had pharmacological prophylaxis, and 132 (13.72%) patients received mechanical prophylaxis. Low molecular weight heparin (LMWH) was used the most for pharmacological prophylaxis. However, both graduated compression stockings (GCS) (*n* = 33, 25.00%) and intermittent pneumatic compression (IPC) (*n* = 31, 23.48%) were used for mechanical prophylaxis. 

### 3.4. Change in In-Hospital HA-VTE Incidents

**Figure 5:**
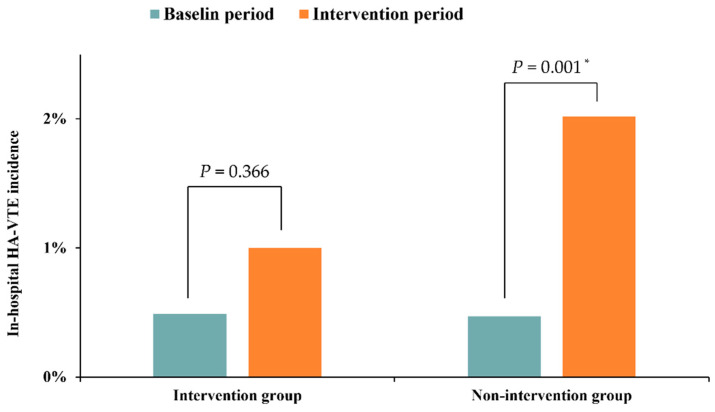
Change in in-hospital HA-VTE Event from baseline to the end of intervention. * *p* < 0.05.

During the baseline period, the intervention group had a total of five hospital-associated VTE events, with an HA-VTE incidence of 0.49%. During the intervention period, a total of six in-hospital HA-VTE events were reported in the intervention group, with an HA-VTE incidence of 1.00%. There was no significant difference in the change in HA-VTE incidence before and after the intervention (*p* = 0.366). The nonintervention group registered a total of 6 HA-VTE events at baseline, with an HA-VTE incidence of 0.47%, and a total of 15 HA-VTE events in the intervention period, with an HA-VTE incidence of 2.02%. For the nonintervention group, HA-VTE incidence increased significantly between the two periods (*p* = 0.001). Figure 5 shows the change of in-hospital HA-VTE events from baseline to the end of intervention. 

## 4. Discussion

Multifaceted quality improvement intervention based on the CDSS is a multidisciplinary collaborative strategy that integrates a series of effective measures. We conducted a pilot study to explore the effect of multifaceted quality improvement intervention on VTE prophylaxis for inpatients. The results of the single-center, department-based, cluster randomized trial showed feasibility for implementation and positive effect on the improvement of VTE prophylaxis with multifaceted quality improvement intervention based on the CDSS, which may provide real-world evidence of multifaceted quality improvement intervention for further development. 

The adoption of the CDSS will help improve quality of healthcare and patient safety, reduce waste in the healthcare system, and reduce the risk of an overwhelming overload for clinicians [18]. Current VTE prophylaxis strategies focus on assessing patients’ VTE risk and bleeding risk and proactively taking the appropriate prophylactic measures based on these risks [8]. Currently, clinically available VTE risk scoring models are generally developed based on European and American evidence. Since the risk factors for acquired VTE in Asian populations are similar to those in European and American populations among hospitalized patients, these models have also been partially validated in Asian populations and are gradually being used in clinical practice in China. In our study, the VTE risk assessment for inpatients was initially performed by the nurses and then confirmed by the physicians. The assessment results were recorded in the nursing system and then automatically sent to the attending physicians’ electronic medical record system for confirmation. The collaboration between nurses and doctors has resulted in a stable and high rate of VTE risk assessment among inpatients.

The VTE risk assessment rate in the intervention group in our study remained largely stable from the baseline period (93.66%) to the intervention period (94.99%). There was a decrease in the VTE risk assessment rate in the general surgery department of the nonintervention group, which could be due to the COVID-19 pandemic (Figure 1). A study analyzing inpatient data before and after the implementation of a VTE risk assessment model (with Padua and IMPROVE risk scales) in hospitals found no significant difference in the incidence of PE and major bleeding among 413 patients, and only 43.3% of patients received pharmacological prophylaxis after the use of the VTE risk assessment scale, compared to 56.7% before [20]. Thus, VTE risk assessment may reduce the medical costs of VTE prophylaxis while keeping patients safe.

In our study, the VTE prophylaxis rate was approximately the same in the intervention and nonintervention groups at baseline. After intervention, the VTE prophylaxis rate increased by 12% in the intervention group with a statistically significant difference, while in the nonintervention group the rate only increased by 5% with no statistically significant difference. Several studies have been conducted to investigate the effectiveness and safety of multiple interventions in VTE prophylaxis. A Cochrane review included 13 RCT studies with a total of 35,997 subjects for analysis, and the results support the conclusion that systematic intervention strategies with proactive reminders can help improve VTE prophylaxis [21]. Kucher et al. included 2506 patients at high risk for VTE and randomized them into two groups with or without electronic alerts. The study found that a significantly greater proportion of patients in the intervention group received mechanical (10.0% vs. 1.5%; *p* < 0.001) or pharmacological prophylaxis (23.6% vs. 13.0%; *p* < 0.001) compared with those in the nonintervention group, and patients in the intervention group had a 41% lower rate of VTE events within 90 days (HR 0.59 [95% CI 0.43–0.81]; *p* = 0.001) [22].

Although our study found no decrease in HA-VTE incidence in the intervention group, a significant increase in the incidence of HA-VTE was found in nonintervention group, revealing an important role of the intervention in limiting the occurrence of HA-VTE. We considered the increase in HA-VTE incidence was mainly due to the limitation of patient activity during the COVID-19 pandemic. Researchers from Johns Hopkins Hospital introduced a mandatory decision support system to facilitate VTE prophylaxis implementation in their hospital information system and enrolled 1599 patients undergoing trauma surgery into their analysis. The study showed that implementation of the mandatory decision support system significantly increased VTE prophylaxis rates in clinical practice (66.2% vs. 84.4%; *p* < 0.001). Moreover, the incidence of HA-VTE decreased significantly after the implementation (1.0% vs. 0.17%; *p* = 0.04) [23]. The University of Virginia Hospital adopted the scheme of Johns Hopkins Hospital and introduced a mandatory CDSS in the implementation of VTE prophylaxis in general surgery patients. The hospital’s 30-day VTE incidence dropped significantly after implementing (1.25% vs. 0.64%; *p* = 0.033), which helped the hospital improve its ranking to the top 10% of 760 hospitals in the National Surgical Quality Improvement Program (NSQIP) in the United States [24]. After participating in the NSQIP, Boston University Hospital also implemented mandatory VTE risk assessment and stratification, and the CDSS automatically recommended the appropriate prophylaxis measures and duration based on the Caprini scores. The result was a significant reduction in the incidence of DVT (from 1.9% to 0.3%) and PE (from 1.1% to 0.5%) in the hospital, highlighting the contribution of the mandatory alert system to the promotion of VTE prophylaxis [25].

We acknowledge that the limited number of participating departments in our study may hinder extrapolation and applicability of the findings, and further validation is needed in studies with larger populations. The department-based cluster randomization in this study can reduce intergroup contamination. The quality control approach of real-time monitoring and reminding through a CDSS enables timely implementation of VTE prophylaxis among multidisciplinary medical staff. Besides the use of different anticoagulant prophylaxis, other efficacy outcomes such as fatal events and VTE events after discharge should also be taken into consideration in evaluating the effect of the VTE prophylaxis in future study [26]. This study forms a pilot study to provide evidence for the feasibility of future trials of multifaceted quality improvement intervention strategies for VTE prophylaxis in multiple centers.

## 5. Conclusions

The multifaceted quality improvement intervention strategies in clinical practice could help improve VTE prevention and reduce the VTE-related safety events in hospitalized patients at risk of VTE. Further study to validation and broader generalization were needed to solve the insufficient VTE prevention in Chinese inpatients.

## Figures and Tables

**Table 1:** Characteristics of patients in baseline and intervention period.

	Baseline Period	Intervention Period ^#^	
		Intervention Group	Nonintervention Group
	(*n* = 2303)	(*n* = 599)	(*n* = 742)
Male	1088 (47.24%)	349 (58.26%)	339 (45.69%)
Age (Years)			
≤40	370 (16.07%)	104 (17.36%)	79 (10.65%)
41–60	758 (32.91%)	180 (30.05%)	219 (29.51%)
61–74	838 (36.39%)	219 (36.56%)	298 (40.16%)
≥75	337 (14.63%)	96 (16.03%)	146 (19.68%)
Medical disease	829 (36.00%)	121 (20.20%)	250 (33.69%)
Malignancy	567 (24.62%)	249 (41.57%)	185 (24.93%)
Surgery	1474 (64.00%)	478 (79.80%)	492 (66.31%)
VTE prophylaxis	548 (23.80%) *	207 (34.56%)	207 (27.90%)
Length of stay (Days)	8	8	8

^#^: Patients in each group were admitted in the same units in both periods. *: no statistical difference of VTE prophylaxis was found between the intervention group and nonintervention group during the baseline period (22.93% vs. 24.49%, *p* = 0.091).

## Data Availability

Data are available upon reasonable request.
